# Integrin β3 Promotes Resistance to EGFR-TKI in Non-Small-Cell Lung Cancer by Upregulating AXL through the YAP Pathway

**DOI:** 10.3390/cells11132078

**Published:** 2022-06-30

**Authors:** Qi Sun, Zhihua Lu, Yanpeng Zhang, Dong Xue, Huayu Xia, Junjun She, Fanni Li

**Affiliations:** 1 Department of General Surgery, The First Affiliated Hospital of Xi’an Jiaotong University, Xi’an 710061, China; sunqi4875@xjtu.edu.cn (Q.S.); x3119315325@stu.xjtu.edu.cn (D.X.); shejunjun@xjtu.edu.cn (J.S.); 2 Department of General Surgery, Qilu Hospital (Qingdao), Cheeloo College of Medicine, Shandong University, Qingdao 266000, China; luzhihua88@mail.sdu.edu.cn; 3 Department of Thoracic Surgery, The First Affiliated Hospital of Xi’an Jiaotong University, Xi’an 710061, China; yanpeng_zhang@xjtu.edu.cn; 4 Xi’an Jiaotong University Health Science Center, Xi’an Jiaotong University, Xi’an 710061, China; xia1045547069@stu.xjtu.edu.cn; 5 Department of Talent Highland, The First Affiliated Hospital of Xi’an Jiaotong University, Xi’an 710061, China

**Keywords:** integrin β3, AXL, NSCLC, EGFR-TKI, drug resistance

## Abstract

Integrin β3 plays a key role in the resistance to epidermal growth factor receptor tyrosine kinase inhibitors (EGFR-TKI), but the development of integrin β3 inhibitors has been stalled due to the failure of phase III clinical trials for cancer treatment. Therefore, it is imperative to find a potentially effective solution to the problem of acquired resistance to EGFR-TKI for patients with integrin-β3 positive non-small-cell lung cancer (NSCLC) by exploring novel downstream targets and action mechanisms of integrin β3. In the present study, we observed that the expression of integrin β3 and AXL was significantly upregulated in erlotinib-resistant NSCLC cell lines, which was further confirmed clinically in tumor specimens from patients with NSCLC who developed acquired resistance to erlotinib. Through ectopic expression or knockdown, we found that AXL expression was positively regulated by integrin β3. In addition, integrin β3 promoted erlotinib resistance in NSCLC cells by upregulating AXL expression. Furthermore, the YAP pathway, rather than pathways associated with ERK or AKT, was involved in the regulation of AXL by integrin β3. To investigate the clinical significance of this finding, the current well-known AXL inhibitor R428 was tested, demonstrating that R428 significantly inhibited resistance to erlotinib, colony formation, epithelial–mesenchymal transformation and cell migration induced by integrin β3. In conclusion, integrin β3 could promote resistance to EGFR-TKI in NSCLC by upregulating the expression of AXL through the YAP pathway. Patients with advanced NSCLC, who are positive for integrin β3, might benefit from a combination of AXL inhibitors and EGFR-TKI therapy.

## 1. Introduction

Lung cancer is the most common malignant tumor globally, with morbidity and mortality ranking first among all malignancies [1]. Non-small-cell lung cancer (NSCLC) comprises about 80% of lung cancer. Surgical treatment can be very effective for patients with NSCLC at an early stage, whereas the majority of patients with NSCLC are originally diagnosed at an advanced stage and may only be treated with medications. Epidermal growth factor receptor (EGFR)-activating mutations occur in approximately 30% to 40% of patients with NSCLC [2], and for these patients EGFR tyrosine kinase inhibitors (TKI) are currently recommended as the standard first-line agents [3]. Despite the inceptive response to EGFR-TKI is impressive, nearly 30% of the responders develop an early relapse within 6 months due to acquired resistance, and almost all the patients eventually relapse [4]. Although a new generation of EGFR-TKI has been developed and applied in clinical practice to counteract some known resistance mechanisms, it seems that such problems as acquired resistance have not been solved efficiently [5]. Multiple mechanisms of acquired resistance to EGFR-TKIs have been characterized, including secondary mutations such as T790M and C797S, activation of alternative signaling pathways and cell phenotypic transformation [6]. Since acquired resistance ultimately limits the clinical efficacy of EGFR-TKI, it is imperative to explore further molecular mechanisms in order to overcome or defer the acquired resistance to EGFR-TKI in patients with NSCLC.

Integrins, a family of cell adhesion receptor proteins, are heterodimer transmembrane glycoproteins comprising α and β subunits, which mainly mediate cell–cell and cell–extracellular matrix adhesion. In addition to functions in physiological development, integrins are involved in the regulation of many cellular functions that are indispensable for cancer progression [7]. Integrin β3, also known as GP3A or CD61, is one of the most extensively studied components of the integrin family. The β3 subunit is only associated with the αv subunit on angiogenetic endothelial cells and tumor cells. As a heterodimer, integrin αvβ3 was detected in a variety of cancers including NSCLC, and its expression was closely related to tumor development and patient prognosis [8]. There is plenty of evidence suggesting that integrin β3 exerts a critical role in the proliferation, migration, metastasis and angiogenesis of tumor cells [9]. In the past few years, a growing body of research has shown that integrin β3 is strongly linked to drug resistance in cancers [10,11,12]. Particularly in NSCLC, integrin β3 inhibits the antitumor activity of ALK inhibitors [13]. Furthermore, integrin β3 is specifically upregulated on the surface of NSCLC cells exposed to EGFR-TKI and is sufficient and necessary to reprogram NSCLC cells to a stemness phenotype with specific resistance to EGFR-TKI [14].

Tyrosine kinases (TKs) family includes major oncoproteins and important mediators of signal transduction that play key roles in cell metabolism, proliferation, differentiation, apoptosis and migration. TKs consist of two main categories, non-receptor tyrosine kinases (NRTKs) and receptor tyrosine kinases (RTKs). AXL, an RTK, plays a variety of functions in normal cells, and its overexpression predicts worse outcomes in various types of human cancers [15]. Overexpression of AXL contributes to tumor progression by facilitating tumor growth, invasion/metastasis, drug resistance and epithelial–mesenchymal transformation (EMT). In NSCLC, the role of AXL in drug resistance has been well studied [16]. The upregulation of AXL may lead to acquired resistance to EGFR-TKI in NSCLC with EGFR mutations [17].

Both integrin β3 and AXL have been already reported to participate in the resistance to EGFR-TKI. However, the resistance mechanisms activated in NSCLC involving the interaction between integrin β3 and AXL remain, to a great extent, unclear. The present study sought to investigate the effect of integrin β3 on AXL expression and the mechanism by which integrin β3 regulates AXL expression and promotes resistance to EGFR-TKI in NSCLC cells.

## 2. Materials and Methods

### 2.1. Immunohistochemistry (IHC)

We performed IHC of tumor biopsies from six patients with advanced NSCLC bearing EGFR mutations, before erlotinib treatment and after erlotinib resistance. The protocol was approved (approval no. 2018-G-106) by the Institutional Medical Ethics Committee of the First Affiliated Hospital of Xi’an Jiaotong University and was in agreement with the Declaration of Helsinki. Written informed consent was obtained from the patients prior to study commencement. Detailed patients’ data are summarized in Supplemental Table S1. After the process of routine deparaffinization, rehydration and antigen retrieval, the tissue slides were incubated with primary anti-integrin β3 (Cell Signaling Technology, Danvers, MA, USA, Cat #13166, RRID: AB_2798136, 1:250) and anti-AXL (Cell Signaling Technology, Cat #8861, RRID: AB_10998619, 1:300) monoclonal antibodies at 4 °C overnight. The next day, horseradish peroxidase-conjugated antibodies and the diaminobenzidine peroxidase substrate were utilized for the establishment of staining.

### 2.2. Assessment of Integrin β3 and AXL Immunostaining

The immunostaining of integrin β3 was mostly presented on the cell membrane of the tumor cells, while AXL was mostly expressed in the cytoplasm. The staining levels were evaluated by two independent observers according to extent and intensity. The staining intensity was scored as 3 (strong), 2 (moderate), 1 (weak) or 0 (none), and the staining extent as 4 (76–100%), 3 (51–75%), 2 (26–50%), 1 (1–25%) or 0 (0%). The IHC score of each NSCLC specimen was decided by multiplying the staining intensity by the extent grade.

### 2.3. Cell Cultures and Transfection

The NSCLC cell lines HCC4006 and HCC827 containing EGFR-activating mutations were purchased from ATCC and cultured in complete RPMI-1640 medium containing 10% fetal bovine serum in a humidified atmosphere with 5% CO_2_ at 37 °C. The cell lines were identified (Cellcook Biotech Company, Guangzhou, China) with STR analysis. Erlotinib (Selleckchem, Houston, TX, USA), an EGFR-TKI, was used to treat NSCLC cells. To obtain erlotinib-resistant cell lines (HCC827 ER and HCC4006 ER), the gradual dosage increase approach was applied, starting with a dosage close to IC50 and increasing it gradually over a period of 6 months. All identified erlotinib-resistant substrains were continuously kept at low doses of erlotinib that allowed cells to proliferate.

Stable cell lines expressing integrin β3 ectopically were established by lentivirus transfection with an empty vector or integrin β3 cDNAs (Addgene Inc., Watertown, MA, USA). Human-specific short hairpin RNAs (shRNA) targeting integrin β3 or control (non-silencing) were assembled into GIPZ vectors (Open BioSystems Inc., Waltham, MA, USA) and then transfected into the cells by a lentivirus to generate cell lines with stable knockdown of integrin β3. The cells were selected with 3 μg/mL of puromycin. Transient overexpression of AXL or YAP was achieved through HA-labeled AXL or YAP cDNA in pCEP4 vector (Addgene Inc., USA) in the indicated cells. FlexiTube siRNAs (QIAGEN, Waltham, MA, USA) included All-Stars negative control and specific siRNAs targeting AXL or YAP. All cDNAs were transiently transfected with Lipofectamine 3000 (Invitrogen, Waltham, MA, USA), and siRNAs with the HiPerFect transfection reagent (QIAGEN, Waltham, MA, USA). The manufacturers’ manuals were strictly followed during the transfection process.

### 2.4. Quantitative Real-Time PCR (qRT-PCR)

Total RNA was extracted from cultured cells by the RNeasy Mini Kit (QIAGEN, Waltham, MA, USA). The High-Capacity cDNA Reverse Transcription Kit (Thermo Fisher Scientific, Waltham, MA, USA) was used to carry out reverse transcription. qRT-PCR was performed with SYBR Green Master Mix (Roche Life Science, Basel, Switzerland). Glyceraldehyde-3-phosphate dehydrogenase (GAPDH) was used as the internal reference. The relative mRNA levels were analyzed with the 2^−ΔΔCt^ method. The sequences of the primers used were: integrin β3 5′-TTCAATGCCACCTGCCTCAA-3′ (forward) and 5′-TTGGCCTCAATGCTGAAGCTC-3′ (reverse); AXL 5′-CGTAACCTCCACCTGGTCTC-3′ (forward) and 5′-TCCCATCGTCTGACAGCA-3′ (reverse); GAPDH 5′-GAGTCAACGGATTTGGTCGT-3′ (forward) and 5′-GACAAGCTTCCCGTTCTCAG-3′ (reverse).

### 2.5. Protein Extraction and Western Blot

Cells were lysed in RIPA (Beyotime, Shanghai, China) with 1% phosphatase inhibitor (Solarbio, Beijing, China) and 1% phenylmethanesulfonyl fluoride (Beyotime, China). Proteins were resolved via 10% sodium dodecyl sulfate polyacrylamide gel electrophoresis and transferred to polyvinylidene fluoride membranes, followed by blocking in 5% skim milk and incubation with primary antibodies at 4 °C overnight. Finally, the membranes were visualized using enhanced chemiluminescence. The primary antibodies applied were: integrin β3 (Cell Signaling Technology, Cat #13166, RRID:AB_2798136, 1:1000), AXL (Cell Signaling Technology, Cat #8861, RRID:AB_10998619,1:1000), YAP (Cell Signaling Technology, Cat #14074, RRID:AB_2650491, 1:1000), E-cadherin (Cell Signaling Technology, Cat #3195, RRID:AB_2291471,1: 1000), Vimentin (Cell Signaling Technology, Cat #5741, RRID:AB_10695459,1: 1000), β-actin (Cell Signaling Technology, Cat #4970, RRID:AB_2223172,1: 1000) and Histone H3 (Cell Signaling Technology, Cat #4499, RRID:AB_10544537,1: 2000).

### 2.6. Preparation of Nuclear and Cytosolic Protein Extracts

The cells were incubated in 500 μL of Buffer A 10× (100 mM KCL, 100 mM EDTA, 100 mM HEPES) with protease inhibitor, 0.5% MP-40 and 1 mM DTT on ice for 10 min. Subsequently, the cells were scraped into a new tube and centrifuged for 15 min at 4 °C at 13,500 RPM. Supernatants containing the cytoplasmic portion were gathered and transferred to a new tube. The nuclear fractions were rinsed with a buffer solution three times, and then diluted with 50 μL of lytic buffer B with a protease inhibitor and 1 mm DTT. The tube containing the nuclear fraction was shaken at 4 °C and centrifuged and then 13,500 RPM for 5 min. The total protein content was measured by the BCA Protein Assay (Pierce, Waltham, MA, USA).

### 2.7. Cell Viability Assay

The CCK-8 kit (Beyotime, China) was used for the cell viability assay. The treated NSCLC cells were seeded into 96-well culture plates (2000 cells/well). After treatment with erlotinib for 48 h, the CCK-8 reagents were added. The absorbance was measured using a Spectra Max 190 instrument (Molecular Devices, San Jose, CA, USA).

### 2.8. Colony Formation Assay

Cells were inoculated into 6-well cell culture plates at 1000 cells/well (HCC827) or 2000 cells/well (HCC4006), and the medium was renewed every 3 days. After 14 days, the colonies were fixed for 10 min in 4% paraformaldehyde, stained for 15 min with a 0.05% crystal violet solution and then counted.

### 2.9. Transwell Migration Assay

Cell migration was assessed using Transwell Boyden chambers (Corning, Corning, NY, USA). In total, 100,000 cells (HCC827) or 150,000 cells (HCC4006) were added to the upper chamber in a volume of 150 μL serum-free medium, and 500 μL of complete medium was added to the lower chamber. After culture for 24 h, the cells were fixed with 4% paraformaldehyde for 10 min and stained with 1% crystal violet for 30 min. The nonmigrating cells attached to the upper surface of the membrane were gently wiped off with a wet cotton swab. The transmembrane cells were counted in five randomly selected high-power visual fields, and their was number averaged.

### 2.10. Statistical Analysis

GraphPad Prism 7 software (San Diego, CA, USA) was used for statistical analysis. Two-tailed Student’s *t*-tests were performed to compare two means, and ANOVAs for data sets with more than three groups. Post-hoc analysis was performed for multiple comparisons. A 2-tailed Wilcoxon matched-pairs test was utilized to compare the immunostaining levels of integrin β3 or AXL in tumor specimens. *p* < 0.05 indicated statistical significance.

## 3. Results

### 3.1. Integrin β3 and AXL Are Significantly Upregulated in Erlotinib-Resistant NSCLC

**Figure 1:**
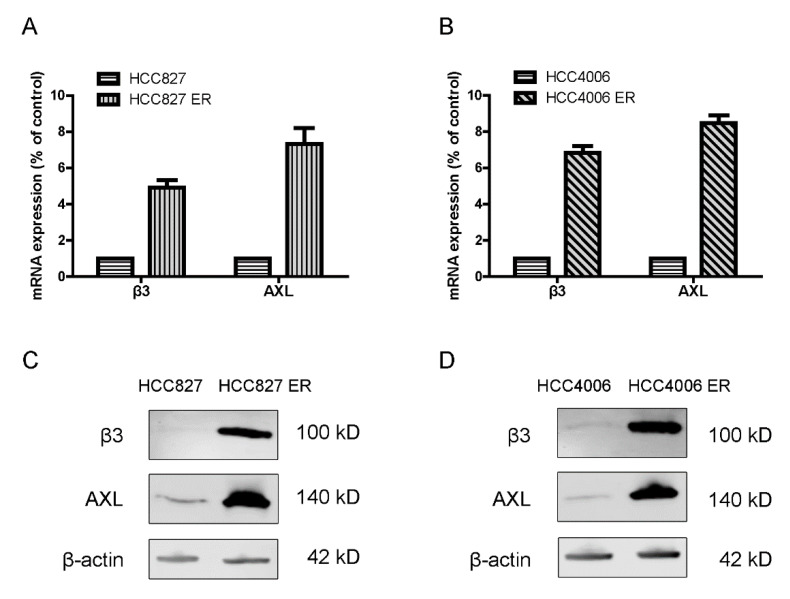
Integrin β3 and AXL were significantly upregulated in erlotinib-resistant (HCC827 ER and HCC4006 ER) cell lines compared with parental (HCC827 and HCC4006) NSCLC cell lines. (**A**,**B**) The mRNA levels of integrin β3 and AXL were determined by qRT-PCR in erlotinib-resistant cells compared with parental cells. GAPDH was used as a loading control. (**C**,**D**) Western blot assays showing integrin β3 and AXL expression in erlotinib-resistant and parental cells. β-actin was used as a loading control. *n* = 3 independent experiments.

To investigate the role of integrin β3 and AXL in EGFR-TKI resistance in NSCLC, we compared the expression of integrin β3 and AXL in erlotinib-resistant and parental NSCLC cell lines. qRT-PCR and western blot assays verified that the levels of both mRNA (Figure 1A,B) and protein expression (Figure 1C,D) of integrin β3 and AXL increased significantly in erlotinib-resistant cells (HCC827 ER and HCC4006 ER) compared with parental cells (HCC827 and HCC4006). 

**Figure 2:**
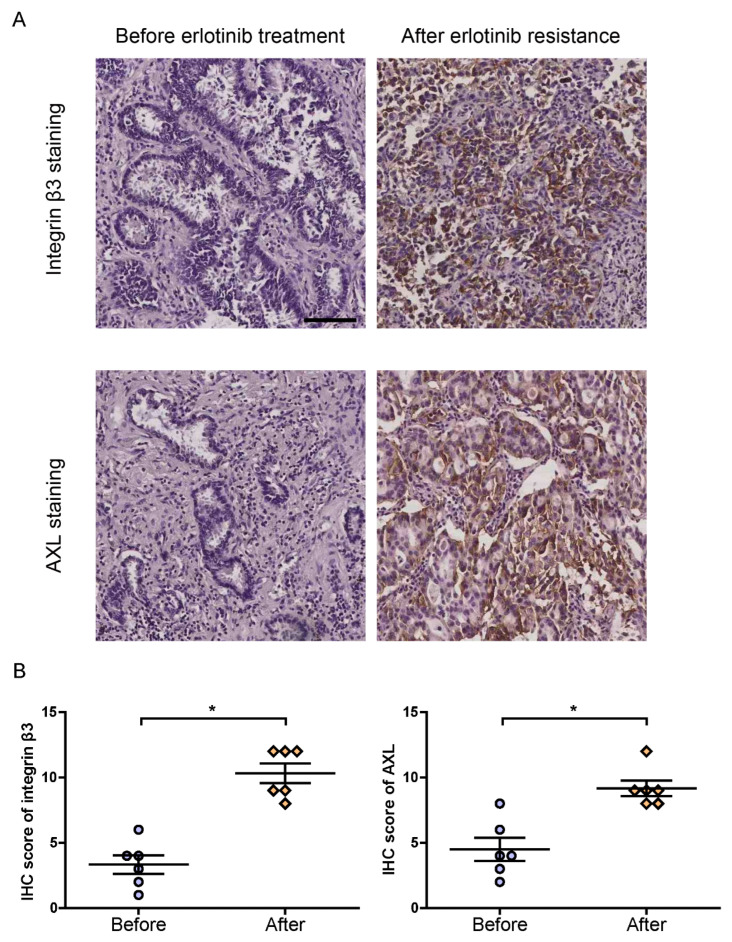
Integrin β3 and AXL were significantly upregulated in NSCLC tissues after the development of acquired erlotinib resistance. (**A**) Representative images of integrin β3 and AXL immunostaining in NSCLC tissues before erlotinib treatment and after acquired erlotinib resistance. (**B**) IHC score demonstrated stronger immunostaining of integrin β3 and AXL in six pairs of NSCLC tissues after the appearance of erlotinib resistance. Data represented the means ± SEM, * *p* < 0.05. Scale bar, 100 μm.

Tumor biopsy samples were collected from six patients before the initiation of erlotinib treatment and after the development of acquired erlotinib resistance. The expression of integrin β3 and AXL was hence further analyzed by IHC, which revealed a higher expression in NSCLC tissues upon establishment of erlotinib resistance (Figure 2A,B). These results indicated that integrin β3 and AXL were significantly upregulated in erlotinib-resistant NSCLC cells (Figure 2). 

### 3.2. Integrin β3 Positively Regulates AXL in NSCLC Cells

**Figure 3:**
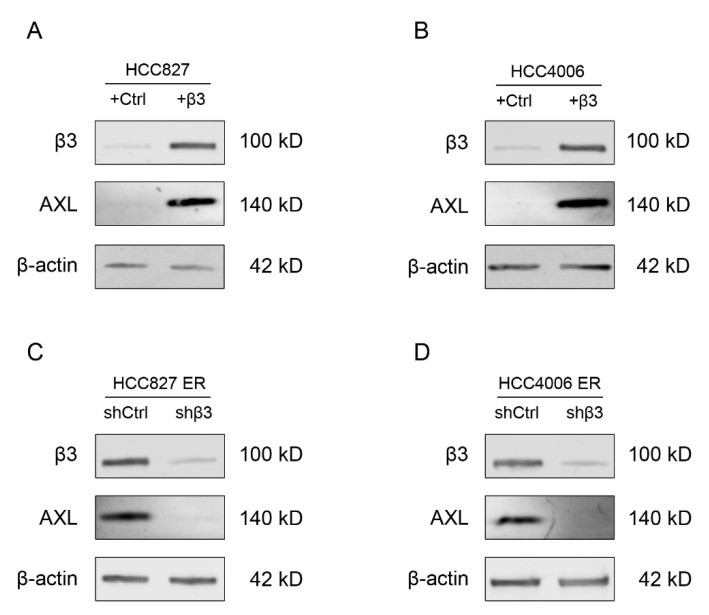
Integrin β3 upregulated AXL in NSCLC cells. Effects of stable ectopic expression of integrin β3 (+β3) or empty vector control (+Ctrl) on AXL expression in HCC827 (**A**) and HCC4006 (**B**) cells. Effects of knockdown of integrin β3 (shβ3) or nonsilencing control (shCtrl) on AXL expression in HCC827 ER (**C**) and HCC4006 ER (**D**) cells. β-actin was used as a loading control. *n* = 3 independent experiments.

To investigate the relationship between integrin β3 and AXL in NSCLC cells, we firstly performed stable ectopic expression of integrin β3 in NSCLC parental cell lines (HCC827 and HCC4006) (Figure 3A,B). Ectopic expression of integrin β3 upregulated AXL expression, while knockdown of integrin β3 in erlotinib-resistant cell lines (HCC827 ER and HCC4006 ER) downregulated AXL expression (Figure 3C,D). These data indicated that integrin β3 could positively regulate AXL expression in NSCLC cells.

### 3.3. Integrin β3 Promotes Erlotinib Resistance by Upregulating AXL in NSCLC Cells

**Figure 4:**
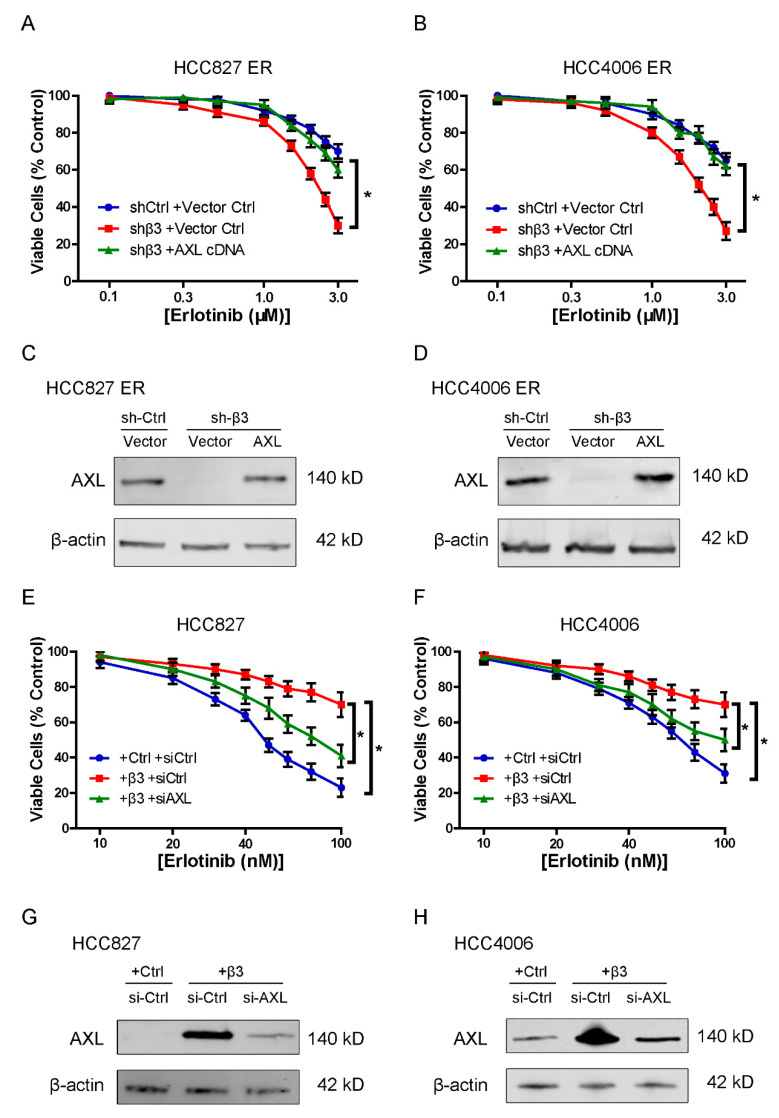
Integrin β3 promoted erlotinib resistance by upregulating AXL in NSCLC cells. (**A**,**B**) CCK-8 assay exploring the effect of ectopic expression of AXL (AXL cDNA) on cell viability in the absence (shβ3) of integrin β3 in HCC827 ER (**A**) and HCC4006 ER (**B**) cells upon treatment with different concentrations of erlotinib. * represents shβ3 +Vector Ctrl vs. shCtrl +Vector Ctrl or shβ3 + AXL cDNA. (**C**,**D**) Western blot assays showing the efficiency of ectopic expression of AXL in integrin β3 knockdown (sh-β3) HCC827 ER (**C**) and HCC4006 ER (**D**) cells. (**E**,**F**) CCK-8 assay exploring the effect of depletion of AXL (si-AXL) on cell viability in the presence (+β3) of integrin β3 in HCC827 (**E**) and HCC4006 (**F**) cells upon treatment with different concentrations of erlotinib. (**G**,**H**) Western blot assays showing the efficiency of AXL depletion in integrin β3-overexpressing (+β3) HCC827 (**G**) and HCC4006 (**H**) cells. (**C**,**D**,**G**,**H**) β-actin was used as a loading control. (**A**,**B**,**E**,**F**) Data represent the means ± SEM, * *p* < 0.05. (**A**–**H**) *n* = 3 independent experiments.

Previous studies indicated that AXL participates in acquired resistance to EGFR-TKI in NSCLC [17]. Hence, we hypothesized that AXL might be involved in integrin β3-mediated erlotinib resistance of NSCLC cells. To investigate the effect of integrin β3 and AXL on the sensitivity of NSCLC cells to erlotinib, the transfected cells were treated with erlotinib at various concentrations. As expected, depletion of integrin β3 led to a higher sensitivity of HCC827 ER cells and HCC4006 ER cells to erlotinib (Figure 4A,B). On the contrary, the sensitivity to erlotinib was reduced in HCC827 cells and HCC4006 cells after overexpression of integrin β3 (Figure 4E,F). More importantly, overexpression of AXL significantly reversed the reduced survival rates of HCC827 ER and HCC4006 ER cells upon erlotinib treatment induced by integrin β3 knockdown (Figure 4A–D). Conversely, knockdown of AXL significantly reversed the improved survival rates upon erlotinib treatment of HCC827 and HCC4006 cells induced by integrin β3 overexpression (Figure 4E–H). Collectively, these findings indicated that integrin β3 promoted erlotinib resistance by upregulating AXL in NSCLC cells.

### 3.4. YAP Is Involved in the Regulation of AXL Expression by Integrin β3 in NSCLC Cells

**Figure 5:**
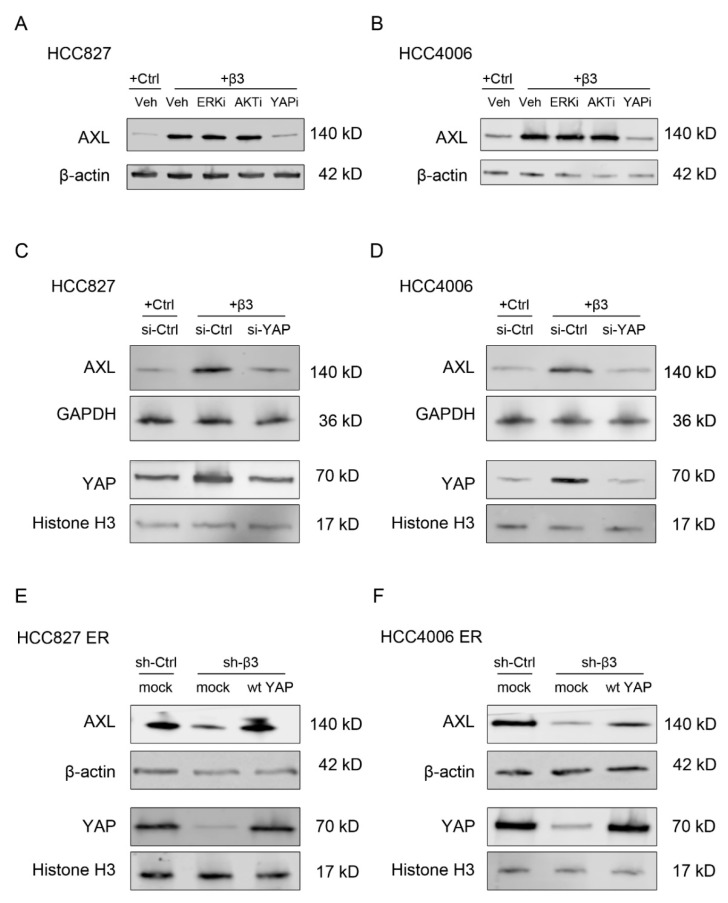
YAP is involved in the regulation of AXL expression by integrin β3 in NSCLC cells. (**A**,**B**) Western blot assays showing the effects of pathway inhibitors on AXL expression after ectopic integrin β3 expression (+β3) in HCC827 (**A**) and HCC4006 (**B**) cells. Veh, Vehicle; ERKi, PD98059; AKTi, LY294002; YAPi, verteporfin. (**C**,**D**) Western blot assays showing the effects of YAP knockdown (si-YAP) on the expression of AXL and nuclear YAP after ectopic integrin β3 expression (+β3) in HCC827 (**C**) and HCC4006 (**D**) cells. (**E**,**F**) Western blot assays showing the effects of YAP activation (wt YAP) on the expression of AXL and nuclear YAP after integrin β3 knockdown (sh-β3) in HCC827 ER (**E**) and HCC4006 ER (**F**) cells. (**A**–**F**) β-actin, GAPDH and Histone H3 were used as loading controls. *n* = 3 independent experiments.

It is well known that integrin β3 plays vital roles in tumor progression and reprogramming of the tumor microenvironment through classical downstream signaling pathways such as PI3K/AKT [18], MEK/ERK [19] and YAP/TAZ [20]. Since YAP has been reported to mediate resistance to EGFR-TKI in NSCLC cells by upregulating the expression of AXL [21], we explored whether it was involved in the regulation of AXL expression by integrin β3. Western blot assays showed that nuclear YAP expression was upregulated in erlotinib-resistant compared with parental cells (Supplemental Figure S1A,B). In addition, the YAP pathway inhibitor verteporfin, rather than ERK and AKT inhibitors, could significantly inhibit AXL expression induced by integrin β3 overexpression (Figure 5A,B). In accordance, overexpression of integrin β3 significantly promoted AXL and nuclear YAP expression, which could be reversed by depletion of YAP in HCC827 and HCC4006 cells (Figure 5C,D). Knockdown of integrin β3 significantly inhibited AXL and nuclear YAP expression, which could be rescued by activation of YAP in HCC827 ER and HCC4006 ER cells (Figure 5E,F), indicating that YAP participated in the regulation of AXL expression by integrin β3 in NSCLC cells (Supplemental Figure S2).

### 3.5. AXL Inhibition Reverses Erlotinib Resistance and Colony Formation Induced by Integrin β3 in NSCLC Cells

**Figure 6:**
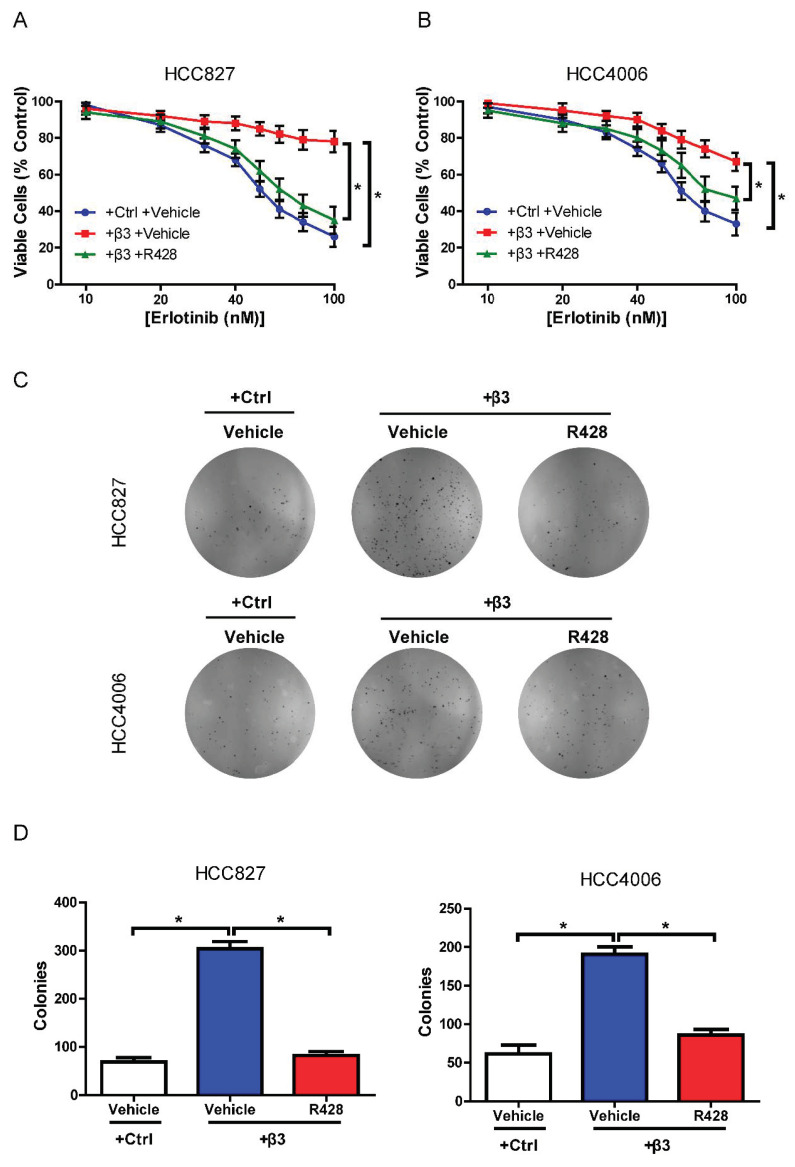
**The** AXL inhibitor R428 reversed erlotinib resistance and colony formation induced by integrin β3 in NSCLC cells. (**A**,**B**) CCK8 assay showing the effects of R428 on cell viability in ectopic integrin β3-expressing (+β3) HCC827 (**A**) and HCC4006 (**B**) cells upon treatment with different concentrations of erlotinib. * represents +β3 +R428 vs. +Ctrl +R428 or +β3 +Vehicle. (**C**,**D**) Colony formation assays showing the effects of R428 on colony formation in ectopic integrin β3-expressing (+β3) HCC827 (**C**) and HCC4006 (**D**) cells. (**A**,**B**,**D**) Data represent the means ± SEM. (**A**–**D**) *n* = 3 independent experiments. * *p* < 0.05.

Given the involvement of AXL in integrin β3-mediated erlotinib resistance, the well-known AXL inhibitor R428 was tested. As a highly selective single-target inhibitor, R428 targets AXL in an ATP-competitive manner. Previous studies have shown that R428 reverses acquired resistance to erlotinib in patients with triple-negative breast cancer [22]. In order to investigate the effect of R428 on acquired resistance to erlotinib induced by ectopic expression of integrin β3, the transfected cells were treated with erlotinib at different concentrations. As expected, R428 significantly reversed the increased survival of HCC827 and HCC4006 cells upon erlotinib treatment induced by ectopic expression of integrin β3 (Figure 6A,B). Moreover, overexpression of integrin β3 promoted colony formation, which could be reversed by R428 in NSCLC cells (Figure 6C,D).

### 3.6. AXL Inhibition Reverses EMT and Migration Induced by Integrin β3 in NSCLC Cells

**Figure 7:**
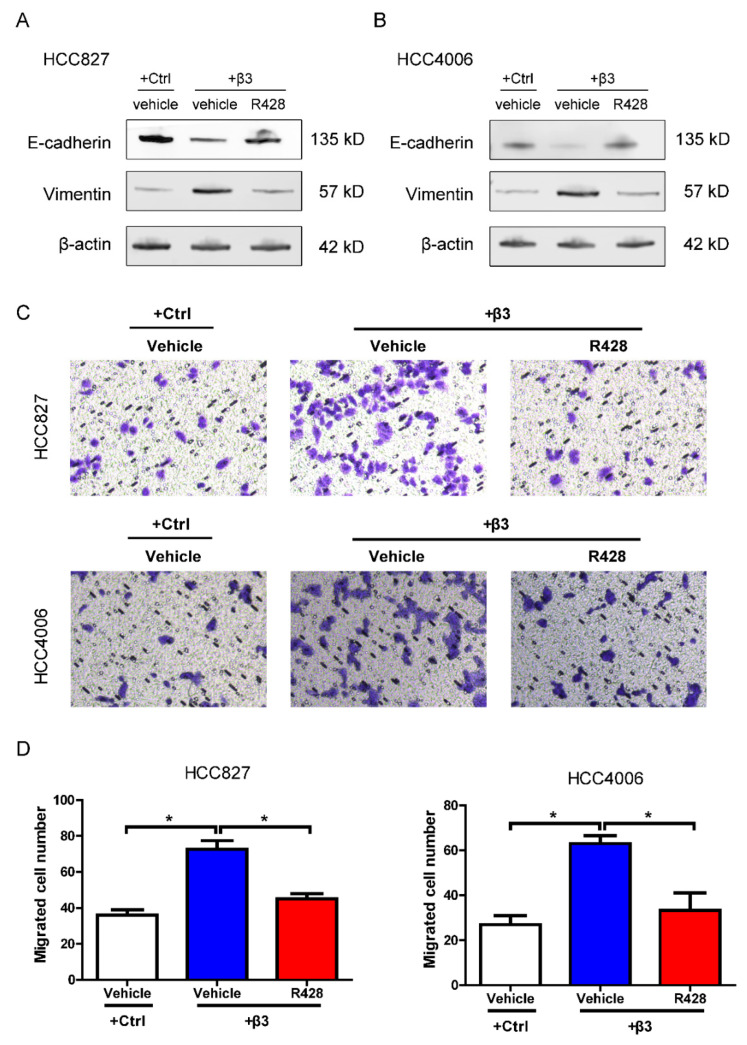
AXL inhibitor R428 reversed EMT and cell migration induced by integrin β3 in NSCLC cells. (**A**,**B**) Western blot assays showing the effects of R428 on E-cadherin and vimentin expression in ectopic integrin β3-expressing (+β3) HCC827 (**A**) and HCC4006 (**B**) cells. (**C**,**D**) Transwell migration assays showing the effects of R428 on cell migration in ectopic integrin β3-expressing (+β3) HCC827 (**C**) and HCC4006 (**D**) cells. (**D**) Data represent the means ± SEM, * *p* < 0.05. (**A**–**D**) *n* = 3 independent experiments.

EMT promotes resistance to targeted therapies and chemotherapeutic agents, which may be an indirect but major mechanism of drug resistance. EMT has been reported to be associated with acquired resistance to EGFR-TKI [23]. We hence explored the effect of AXL on EMT induced by integrin β3. As expected, ectopic expression of integrin β3 induced vimentin expression and reduced E-cadherin expression, effects that were reversed by R428 (Figure 7A,B). In addition, R428 could suppress cell migration promoted by the overexpression of integrin β3 (Figure 7C,D). These results suggested that AXL inhibition could reverse EMT and cell migration induced by integrin β3 in NSCLC cells.

## 4. Discussion

As a cell membrane adhesion receptor, integrin β3 takes part in shaping the stromal and immune microenvironment, reprogramming tumor metabolism, promoting endothelial to mesenchymal transition (End-MT) and EMT, and maintaining tumor stemness. Drug resistance is an important characteristic of malignancy that contributes to high recurrence and mortality rates. It has been reported that integrin β3 overexpression is associated with drug resistance and can predict poor prognosis in patients with NSCLC [14]. AXL has been identified to facilitate intrinsic and acquired resistance to molecularly targeted, immunotherapeutic and chemotherapeutic agents in malignancies [24]. In EGFR TKI-resistant tumor xenografts, the expression level of AXL was found to be elevated, and inhibition of AXL recovered the sensitivity to EGFR-TKI, which implies that activation of AXL is essential for acquired resistance to EGFR-TKI in NSCLC [25]. A previous study reported that integrin β3 could crosstalk with AXL under physiological conditions and exerted an anti-apoptotic effect on vascular endothelial cells [26]. Yet, the relationship between integrin β3 and AXL in tumors has been reported only in one study on high-grade ovarian cancer, in which Gas6/AXL signaling cooperated with the integrin β3 pathway via the adaptor protein p130Cas, contributing to the adhesion and invasion of epithelial ovarian cancer cells [27]. Based on the above clues, we speculated that integrin β3 may play an important role in acquired resistance to EGFR-TKI in NSCLC by regulating AXL.

In the present study, to validate our conjecture, immunohistochemical staining was used to detect the expression levels of integrin β3 and AXL in cancer biopsy samples before erlotinib treatment and after acquired erlotinib resistance in patients with NSCLC. Surprisingly, both integrin β3 and AXL levels were significantly increased after the establishment of erlotinib resistance. Consistently, the expression levels of integrin β3 and AXL were significantly upregulated in erlotinib-resistant cells compared with parental NSCLC cells. To further investigate whether integrin β3 could regulate AXL expression in EGFR-TKI resistant cells, integrin β3 was knocked down in erlotinib-resistant NSCLC cell lines, which subsequently led to a significant reduction of AXL expression as well as a decreased resistance to erlotinib. What is more important, this effect could be rescued by the overexpression of AXL. In contrast, ectopic expression of integrin β3 in NSCLC parental cells upregulated AXL and significantly enhanced the resistance to erlotinib. The above results suggest that integrin β3 increased the resistance to EGFR-TKI in NSCLC cells by upregulating the expression of AXL.

Several classical pathways including AKT, ERK and YAP have been reported to participate in cancer development mediated by integrin β3. In our study, we found that inhibition of YAP, rather than of AKT or ERK pathways, significantly downregulated AXL expression. YAP is a transcriptional coactivator that shuttles between nucleus and cytoplasm, reacting to various inputs in Hippo-dependent and non-Hippo-dependent pathways. In the nucleus, YAP combines with DNA-binding factors of the TEAD family to regulate gene expression, promoting cell proliferation, stress survival, organ overgrowth, and post-mitotic cell dedifferentiation into tissue progenitor cells [28]. The PAK4/YAP pathway is the main downstream signaling pathway of integrin β3 driving Glut3 expression to promote glioblastoma progression [20]. Inhibition of the Hippo pathway effector YAP restores sensitivity to EGFR-TKI following primary or acquired resistance to EGFR-TKI in NSCLC [29]. In addition, acquired resistance to EGFR-TKIs in NSCLC is dependent on the expression and nuclear localization of YAP [30]. Moreover, YAP has been reported to mediate resistance to EGFR-TKI in NSCLC cells by upregulating the expression of AXL [21]. Therefore, we further examined the effect of integrin β3 on AXL expression by depletion or activation of YAP. We found that in erlotinib-resistant NSCLC cell lines, knockdown of integrin β3 significantly reduced expression levels of AXL and nuclear YAP, while overexpression of YAP significantly rescued the decreased expression of AXL. As expected, in wild-type NSCLC cell lines, ectopic expression of integrin β3 significantly increased the expression of AXL and nuclear YAP, while knockdown of YAP significantly reversed the increased expression of AXL. The above findings suggest that the YAP pathway is involved in integrin β3-mediated upregulation of AXL in NSCLC. 

Given the multiple roles of integrins β3 in tumor progression and metastasis, many inhibitors such as Cilengitide, MK-0429 and Vitaxin have been subjected to preclinical and clinical trials. Notably, Cilengitide, a competitive inhibitor of ligand binding to integrin αvβ3, is an antiangiogenic agent of choice. Although it showed encouraging results in phase I/II clinical trials in several kinds of solid tumors including glioblastoma, lung cancer and melanoma [31], phase III trials failed to show a significant improvement in patients’ prognosis [32,33,34], suggesting complexity of the mechanisms of action of integrin β3 in malignancies. It is important to note, however, that although the primary function of integrins is the coordination of cell–matrix communication which influences intracellular signaling cascades [35], integrin β3 is able to trigger anchorage-independent cell survival and tumor metastasis in the absence of ligand binding [36], which may explain to a certain degree the failure of Cilengitide in clinical trials. Specifically, integrin β3 is able to recruit KRAS and RalB to the cell membrane in an unliganded state, leading to the activation of TBK1 and NF-κB, thus driving tumor stemness and resistance to EGFR-TKI in NSCLC [14].Therefore, a full understanding of the molecular mechanisms of integrin β3 in drug resistance would be helpful to directly design specific targeting strategies. By investigating the mechanism of EGFR-TKI resistance induced by integrin β3, we aimed to provide an alternative strategy to obviate to the current situation of failed clinical trials of integrin β3 inhibitors. Due to the high selectivity of R428, induction of apoptosis in the absence of AXL is limited, which provides R428 with the potential advantage of causing less side effects in cancer therapy. Phase I/II clinical trials of R428 have been conducted in patients with metastatic breast cancer, acute myeloid leukemia, and NSCLC [37]. In our study, R428 inhibited erlotinib resistance, colony formation, EMT and migration induced by integrin β3 in NSCLC cells. It is promising that R428 may be approved for clinical use in the future. In the context of the multi-targets AXL inhibitors currently being developed and widely used in clinical practice [38], our results provide theoretical evidence for a combination therapy including AXL inhibitors to treat integrin β3-positive NSCLC patients receiving EGFR-TKI. Still, further work is needed to draw more convincing conclusions. Additionally, various mechanisms of acquired resistance to EGFR-TKI may act independently or crosstalk; therefore, the relationship between integrin β3, AXL and other known resistance mechanisms such as T790M require further studies in the future.

In conclusion, integrin β3 promotes resistance to EGFR-TKI in NSCLC by upregulating the expression of AXL through the YAP pathway. Patients with advanced NSCLC bearing EGFR mutations and positive for integrin β3 may benefit from a combination of AXL inhibitors and EGFR-TKI therapy.

## Data Availability

The datasets supporting the conclusions of this article are included within the article.

## Supplementary Materials

The following are available online at https://www.mdpi.com/article/10.3390/cells11132078/s1, Figure S1. YAP was activated in erlotinib-resistant (HCC827 ER and HCC4006 ER) cell lines compared with parental (HCC827 and HCC4006) NSCLC cell lines; Figure S2. Semi-quantitative analyses for the western blots in Figure 5; Table S1. Detailed patients’ data of the six patients with advanced NSCLC bearing EGFR mutations who developed erlotinib resistance.

## Abbreviations

NSCLC	Non-small-cell lung cancer
EGFR-TKI	Epidermal growth factor receptor tyrosine kinase inhibitors
EGFR	Epidermal growth factor receptor
TKI	Tyrosine kinase inhibitors
TKs	Tyrosine kinases
NRTKs	Non-receptor tyrosine kinases
RTKs	Receptor tyrosine kinases
EMT	Epithelial–mesenchymal transition
IHC	Immunohistochemistry
shRNA	Short hairpin RNA
siRNA	Small interfering RNA
STR	Short tandem repeat
RIPA	Radio immunoprecipitation assay
RPM	Revolutions per minute
DTT	Dithiothreitol
BCA	Bicinchoninic acid
qRT-PCR	Quantitative real-time PCR
End-MT	Endothelial to mesenchymal transition
ALK	Anaplastic lymphoma kinase
YAP	Yes-associated protein
R428	Bemcentinib
GP3A	Platelet glycoprotein IIIa
CD61	Cluster of differentiation 61
ER	Erlotinib-resistant
CCK-8	Cell counting kit-8
WT	Wild type
PI3K	Phosphatidylinositol 3-kinase
MEK	Mitogen-activated protein kinase
TAZ	Transcriptional co-activator with PDZ-binding motif
