# Supplementary material for: Integrin β3 Promotes Resistance to EGFR-TKI in Non-Small-Cell Lung Cancer by Upregulating AXL through the YAP Pathway
Source: Cells. 2022 Jun 30;11(13):2078. doi: 10.3390/cells11132078 (PMC9265629; doi:10.3390/cells11132078)
Supplement: Supplementary file 1 [file cells-11-02078-s001.zip › cells-1661031-supplementary.pdf]

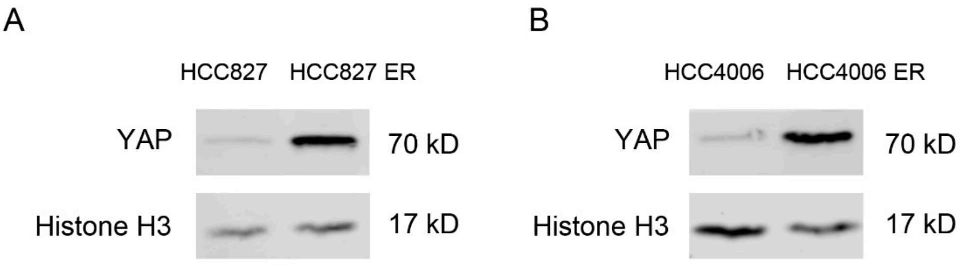

Figure S1. YAP was activated in erlotinib-resistant (HCC827 ER and HCC4006 ER) cell lines compared with parental (HCC827 and HCC4006) NSCLC cell lines. (**A** and **B**) Western blot assays showing nuclear YAP expression in erlotinib-resistant and parental cells. Histone H3 was used as a loading control.

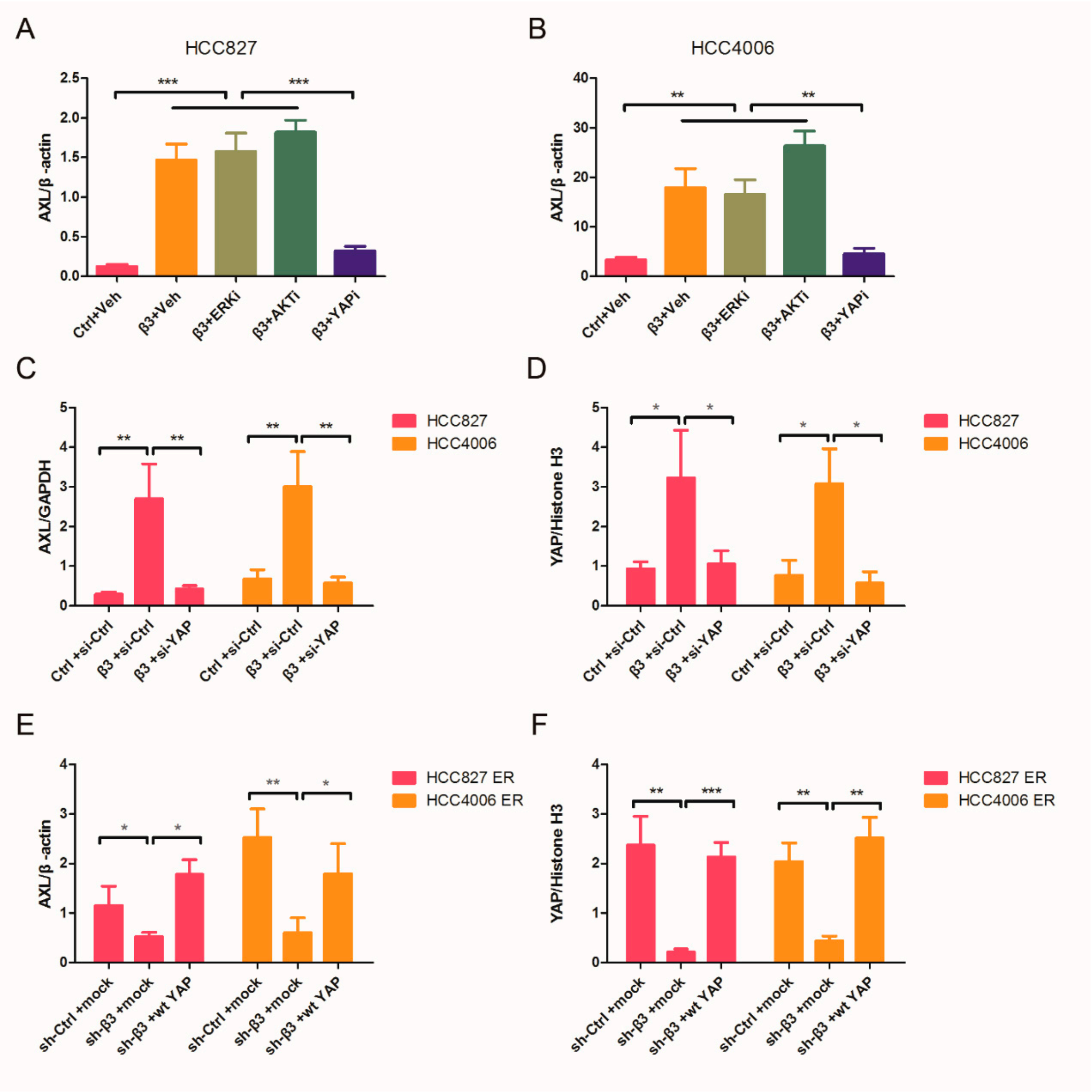

Figure S2. Semi-quantitative analyses for the western blots in Figure 5. (**A** and **B**) Histogram with the fold change showing the effects of pathway inhibitors on AXL expression after ectopic integrin β3 expression (+β3) in HCC827 (**A**) and HCC4006 (**B**) cells seen in Figure 5 A and B. Veh, Vehicle; ERKi, PD98059; AKTi, LY294002; YAPi, verteporfin. (**C** and **D**) Histogram with the fold change showing the effects of YAP knockdown (si-YAP) on the expression of AXL and nuclear YAP after ectopic integrin β3 expression (+β3) in HCC827 (**C**) and HCC4006 (**D**) cells seen in Figure 5 C and

D. (**E** and **F**) Histogram with the fold change showing the effects of YAP activation (wt YAP) on the expression of AXL and nuclear YAP after integrin β3 knockdown (sh-β3) in HCC827 ER (**E**) and HCC4006 ER (**F**) cells seen in Figure 5 E and F. Data represents the means ± SEM, * $p < 0.05$, ** $p < 0.01$, *** $p < 0.001$.

Table S1. Detailed patients' data of the six patients with advanced NSCLC bearing EGFR mutations who developed erlotinib resistance.

Patient	Sex	Age (years)	Tumor type	Smoking status	Treatment period of erlotinib (days)
Patient #1	Male	65	Adenocarcinoma	Yes	200
Patient #2	Female	49	Adenocarcinoma	No	136
Patient #3	Male	59	Adenocarcinoma	Yes	430
Patient #4	Female	62	Adenocarcinoma	No	245
Patient #5	Male	68	Adenocarcinoma	No	310
Patient #6	Male	74	Adenocarcinoma	Yes	95
